# Association between plasma CTRPs with cognitive impairment and neurodegeneration of Alzheimer's disease

**DOI:** 10.1111/cns.14606

**Published:** 2024-02-09

**Authors:** Xiao Huang, Jialing Zhao, Qinghua Wang, Tingqi Yan, Shu Gou, Xiaofeng Zhu, Liu Yang, Fang Ye, Jie Zhang, Yanjiang Wang, Shaojie Yang, Weidong Le, Yang Xiang

**Affiliations:** ^1^ Institute of Neurology, Sichuan Provincial People's Hospital, School of Medicine University of Electronic Science and Technology of China Chengdu China; ^2^ Department of Neurology, Sichuan Provincial People's Hospital, School of Medicine University of Electronic Science and Technology of China Chengdu China; ^3^ Department of Neurology Yunyang County People's Hospital Chongqing China; ^4^ Department of Neurology and Center for Clinical Neuroscience, Daping Hospital Third Military Medical University Chongqing China; ^5^ Chongqing Key Laboratory of Ageing and Brain Diseases Chongqing China; ^6^ Department of Neurology Chengdu Eighth People's Hospital Chengdu China

**Keywords:** Alzheimer's disease, biomarker, cognitive impairment, CTRP, tau protein

## Abstract

**Aims:**

Recent evidence indicated the biological basis of complement 1q (C1q)/tumor necrosis factor (TNF)‐related protein (CTRP) 3, 4, and 14 for affecting brain structure and cognitive function. Thus, we aimed to investigate the association between plasma CTRPs with Alzheimer's disease (AD).

**Methods:**

A multicenter, cross‐sectional study recruited patients with AD (*n* = 137) and cognitively normal (CN) controls (*n* = 140). After the data collection of demographic characteristics, lifestyle risk factors, and medical history, plasma levels of tau phosphorylated at threonine 217 (pT217), pT181, neurofilament light (NfL), CTRP3, 4, and 14 were examined and compared. Multivariate logistic regression analysis was applied to determine associations of plasma CTPRs with the presence of AD. The correlation analysis was used to explore correlations between plasma CTPRs with scores of Mini‐Mental State Examination (MMSE), Montreal Cognitive Assessment (MoCA), Activities of Daily Living (ADL) scale, and Clinical Dementia Rating Sum of Boxes (CDR‐SB), and levels of plasma pT217, pT181, and NfL. Receiver‐operating characteristic (ROC) analysis and Delong's test were used to determine the diagnostic power of plasma CTPRs.

**Results:**

Plasma levels of CTRP3, 4, and 14 were higher in AD group than those in CN group. After adjusting for conventional risk factors, CTRP3, CTRP4, and CTRP14 were associated with the presence of AD. In AD patients, CTRP3 was negatively correlated with scores of MMSE and MoCA, while positively correlated with ADL score, CDR‐SB score, pT217, and pT181; CTRP4 was positively correlated with CDR‐SB score, pT181, and NfL; CTRP14 was negatively correlated with MMSE score, while positively correlated with CDR‐SB score, pT217, and NfL. An independent addition of CTRP3 and 4 to the basic model combining age, sex, years of education, *APOE4* status, BMI, TG, and HDL‐C led to a significant improvement in diagnostic power for AD, respectively.

**Conclusions:**

All the findings preliminarily uncovered associations between plasma CTRPs and AD and suggested the potential of CTRPs as a blood‐derived biomarker for AD.

## Graphical abstract

Plasma levels of three CTRPs members were higher in the AD group than those in the CN group, and were remarkably associated with the presence of AD, suggesting that CTRPs, as a newly discovered adipokine family, have the potential as a blood‐derived biomarker for AD.

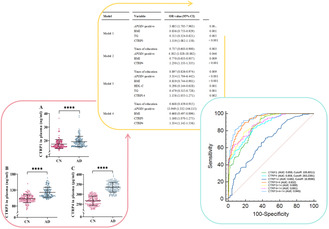

## 1 INTRODUCTION

Alzheimer's disease (AD) is an age‐related neurodegenerative disorder characterized by progressive cognitive and social dysfunctions in clinical manifestations and extracellular plaques formed by β‐amyloid (Aβ) protein and intracellular neuronal fiber tangles formed by tau protein in representative pathological features.^1^, ^2^ As the leading cause of dementia, AD accounts for approximately 60–80% of all dementia cases and has quickly become one of the most expensive, lethal, and worrisome healthcare burdens in this century.^3^, ^4^ It is widely believed that AD is associated with multiple factors such as age, sex, genetics, and environment, but the exact pathogenesis has not been fully elucidated so far.^5^

Previously, we proposed the concept of the brain–visceral adipose tissue axis and preliminarily analyzed its potential influence on the development of AD, in which adipokines secreted by adipose tissue may play multiple regulatory roles in Aβ metabolism, tau phosphorylation, hippocampal neurogenesis, synaptic plasticity, and neuroinflammation.^6^ Of note, complement 1q (C1q)/tumor necrosis factor (TNF)‐related proteins (CTRPs) are a newly discovered adipokine family and are mainly secreted by the adipose tissue.^7^ CTRPs have a common characteristic structure including four different domains, one of which is homologous to adiponectin.^8^ To date, 15 members (CTRP1‐15) have been subsequently termed and their relationships to various diseases have been uncovered; nevertheless, whether CTRPs are correlated with AD has not been reported.

Mounting evidence has proved complicated associations of CTRPs with the brain. CTRP3 has the capacity of freely penetrating the blood–brain barrier (BBB) independent of glucose and lipid metabolism in vivo.^9^ The overexpression of CTRP3 attenuated neuronal apoptosis and neuroinflammation, and improved learning and memory abilities in elderly rats anesthetized with sevoflurane.^10^ Repetitive transcranial magnetic stimulation alleviated learning and memory impairment in rats receiving middle cerebral artery occlusion (MCAO) by activating the CTRP3‐signaling pathway.^11^ In addition to the adipose tissue, CTRP4 is synthesized and secreted by neurons at an early stage of brain development and forms a variety of oligomeric complexes widely distributed in brains of humans, mice, and zebrafish.^12^, ^13^, ^14^, ^15^ Lacking of CTRP4 impaired hippocampal‐dependent associative learning and memory abilities in mice in the fear‐conditioning paradigm by altering expressions of cognition‐related genes in hippocampus and cortex.^13^ CTRP14 generated by climbing fibers acts as a key anterograde signal‐regulating synaptic formation and maintenance,^15^ and is closely related to learning, memory, and synaptic conduction of mice.^16^

All the reports about CTRP3, 4, and 14 above suggest potential associations between CTRPs and a range of neurological diseases. Given that cognitive impairment is the core symptom, and neuroinflammation, neuronal loss, and synaptic degeneration are pathological features of AD, we designed a retrospective study, established both AD and cognitively normal (CN) control groups, explored relationships of plasma CTRP3, 4, and 14 with the presence, main clinical characteristics, and current representative blood biomarkers of AD, and investigated their performances in the diagnosis of this disease.

## 2 MATERIALS AND METHODS

### 2.1 Study design and subject selection

All subjects were recruited from the outpatient departments of neurology of Sichuan Provincial People's Hospital, Chengdu Eighth People's Hospital, and Yunyang County People's Hospital from January 2022 to January 2023 (Figure S1). The diagnosis of AD was in accordance with the criteria for “Probable AD” of the National Institute of Neurological and Speech Disorders and Stroke/Alzheimer's Disease and Related Disorders Association criteria (NINCDS‐ADRDA).^17^ The subjects were excluded if they had:^18^ (1) familial dementia; (2) severe pulmonary, cardiac, hepatic, renal diseases, or any kind of tumor; (3) enduring mental illness (e.g., schizophrenia); or (4) other kinds of cognitive deficits, such as vascular dementia, dementia with Lewy bodies, frontotemporal dementia, dementia of Parkinson's disease, and normal pressure hydrocephalus.

The cognitive state of all subjects was first screened using the China version of the Mini‐Mental State Examination (MMSE) and Montreal Cognitive Assessment (MoCA). Cutoff values after correcting years of education were employed in the MMSE (≤24 points for more than 6 years of education, ≤20 points for less than 6 years of education, and ≤17 points for 0 year of education) and MoCA (≤26 points for more than 12 years of education and ≤25 points for <12 years of education).^18^, ^19^ The Chinese version of the activities of daily living (ADL) scale which included a physical self‐maintenance scale and instrumental activities of daily living scale was used to evaluate the daily functional status of all subjects.^20^, ^21^ Its maximum score is 56 points with a higher score indicating the more impairment of daily living function. The vascular factors were assessed by the Hachinski Ischemic Score (HIS). Subjects with abnormal cognitive function assessment were further evaluated with the Clinical Dementia Rating (CDR) in which patients with AD should have a CDR score ≥ 0.5.^22^ These procedures were administered by two trained interviewers who were experienced neurologists in outpatient departments of neurology. The study was approved by the Ethical Review Board of Sichuan Provincial People's Hospital (No. 2021LSY054) and was registered on the Chinese Clinical Trial Registry (ChiCTR) as ChiCTR2200055620.

Subsequently, baseline data on demographic characteristics (age, sex, and years of education), body mass index (BMI), lifestyle risk factors (cigarette smoking and alcohol consumption), and medical history (diabetes mellitus, hypertension, hyperlipidemia, and coronary heart disease) were collected within 24 h of hospital admission.

### 2.2 Biochemical and molecular investigations

Following our previously published protocols,^18^ fasting blood samples were collected between 6:00 a.m. and 7:00 a.m. within 24 h of admission to avoid variations related to possible circadian rhythm effects, aliquots were stored for routine blood tests [i.e., fasting blood glucose, hemoglobin A1C (HbA1C), total cholesterol (TC), triglyceride (TG), high‐density lipoprotein cholesterol (HDL‐C), and low‐density lipoprotein cholesterol (LDL‐C)], and *apolipoprotein* (*APO*) *E4* genotyping (determined using a Sanger‐sequencing assay for single‐nucleotide polymorphism *rs7412* and *rs429358*) was conducted uniformly in the department of clinical laboratory of Sichuan Provincial People's Hospital.^18^, ^23^

The plasma was separated and frozen at −80°C until measurements of tau phosphorylated at threonine (pT) 217, pT181, neurofilament light chain (NfL), and CTRPs. In accordance with our previous study, the plasma levels of pT217, pT181, and NfL were simultaneously measured using the commercially available single‐molecule array (SIMOA) Human Neurology 3‐Plex A assay kit (Quanterix, USA) on board of the automated SIMOA HD‐1 analyzer (Quanterix).^24^ Plasma levels of CTRP3 (SEK169Hu, Cloud‐Clone Corp., Hubei, China), CTRP4 (MM‐1640H1, Jiangsu Meimian Industrial Co., Ltd., Jiangsu, China), and CTRP14 (SEE867Hu, Cloud‐Clone Corp.) were detected using commercially available ELISA kits according to the instructions.

### 2.3 Statistical analyses

All statistical analyses were performed using IBM SPSS Statistics version 25 (IBM, Armonk, NY, USA) and GraphPad Prism version 8 (GraphPad Software, La Jolla, CA, USA). For continuous variables, the normality of the distribution was first determined by normality diagnostic graphs (histograms, Q–Q plots, and P–P plots) and Kolmogorov–Smirnov test. Then, they were expressed as mean ± standard deviation (SD) or median and interquartile range (IQR) according to results of the normality test. Furthermore, the differences between two groups were compared using *t*‐test or Mann–Whitney *U* test depending on data distribution and homogeneity of variance. Categorical variables were expressed as proportions, and a chi‐square test was applied in the comparison between groups. The influence of plasma CTRPs on the presence of AD was determined by multivariate logistic regression analysis, which allowed adjustment for the confounding factors. Clinical variables with *p <* 0.05 on univariate analysis and the general AD risk factors were incorporated into multivariate logistic regression analysis models. Results were expressed as odds ratio (OR) with corresponding 95% confidence interval (CI). Nominally correlations between plasma CTRPs with clinical characteristics and the representative blood‐based biomarkers of AD were analyzed using Spearman's rank correlation or Pearson's correlation without the adjustment for multiplicity. The clinical value of adding CTRPs to the general risk factors for identifying or predicting the presence of AD was calculated with receiver operating characteristic (ROC) curves. Using DeLong's test, areas under the curve (AUC) between different models were compared. All tests were two‐sided, and *p <* 0.05 was considered to be statistically significant. Goodness‐of‐fit of logistic regression models was assessed using the Hosmer–Lemeshow test. None of the above models displayed a Hosmer–Lemeshow chi‐squared value yielding a *p <* 0.05, and therefore, none were rejected.

## 3 RESULTS

### 3.1 Comparison of baseline data between two groups

A total of 277 subjects were recruited including CN group (*n* = 140) and AD group (*n* = 137), and the detailed enrollment process for subjects is shown in Figure S1. The demographic characteristics, BMI, lifestyle risk factors, medical history, cognitive function, daily living abilities, and blood tests of all subjects are shown in Table 1.

**TABLE 1:** Demographics, clinical characteristics, and blood tests of all subjects.

Variable	CN group (*n* = 140)	AD group (*n* = 137)	*t*/*x* ^2^/*u*	*p* Value
Age, mean (SD), (years)	74.06 (8.26)	75.34 (8.20)	8747	0.2059
Sex, female, No. (%)	72 (51.43)	86 (62.77)	3.64	0.0565
Years of education, median (IQR), (years)	9 (8–12)	6 (5.5–9)	6968	<0.0001
*APOE4*‐positive, No. (%)	24 (17.14)	58 (42.34)	21.09	<0.0001
BMI, mean (SD), (kg/m^2^)	23.86 (3.52)	21.35 (3.15)	6.25	<0.0001
Cigarette smoking, No. (%)	28 (20.00)	29 (21.17)	0.06	0.8100
Alcohol consumption, No. (%)	25 (17.86)	26 (18.98)	0.06	0.8098
Diabetes mellitus, No. (%)	31 (22.14)	39 (28.47)	1.47	0.2259
Hypertension, No. (%)	60 (42.86)	68 (49.64)	1.28	0.2579
Hyperlipidemia, No. (%)	72 (51.43)	57 (41.61)	2.69	0.1013
Coronary heart disease, No. (%)	31 (22.14)	30 (21.90)	0.002	0.9608
Fasting blood glucose, median (IQR)	5.34 (4.62–6.12)	5.06 (4.44–5.94)	1.457	0.1464
HbA1c, median (IQR)	5.80 (5.40–6.38)	5.70 (5.40–6.28)	9128	0.4879
TC, mean (SD), (mmol/L)	4.56 (1.16)	4.40 (1.21)	1.18	0.2382
TG, mean (SD), (mmol/L)	1.78 (1.36)	1.30 (0.51)	7887	0.0106
HDL‐C, mean (SD), (mmol/L)	1.63 (0.47)	1.42 (0.41)	4.31	<0.0001
LDL‐C, mean (SD), (mmol/L)	2.51 (0.85)	2.50 (0.90)	0.07	0.9458
MMSE score, median (IQR)	28 (27–29)	13 (9–19)	115	<0.0001
MoCA score, median (IQR)	28 (26–29)	8 (4–13)	0	<0.0001
ADL score, median (IQR)	16 (16–16)	35 (25–48)	10	<0.0001
CDR global (0/0.5/1/2/3)	140/0/0/0/0	0/5/46/37/49	–	–
CDR‐SB, median (IQR)	0 (0–0)	12 (7–16)	0	<0.0001
pT217, mean (SD), (pg/mL)	181.54 (73.19)	239.15 (120.84)	7223	0.0003
pT181, mean (SD), (pg/mL)	1.69 (0.72)	2.34 (1.04)	6123	<0.0001
NfL, mean (SD), (pg/mL)	657.97 (282.27)	839.04 (371.09)	4.583	<0.0001
CTRP3, mean (SD), (ng/mL)	102.84 (12.60)	122.70 (15.60)	3025	<0.0001
CTRP4, mean (SD), (pg/mL)	267.30 (23.35)	335.42 (24.49)	23.70	<0.0001
CTRP14, mean (SD), (ng/mL)	17.43 (3.31)	19.27 (3.90)	6590	<0.0001

*Note*: Statistical methods: BMI, Fasting blood glucose, TC, HDL‐C, LDL‐C, NfL, and CTRP4 were compared using *t*‐test; Age, Years of education, HbA1c, TG, MMSE score, MoCA score, ADL score, CDR‐SB, pT217, pT181, CTRP3, and CTRP14 were compared using Mann–Whitney *U* test; and Sex, *APOE4*‐positive, Cigarette smoking, Alcohol consumption, Diabetes mellitus, Hypertension, Hyperlipidemia, and Coronary heart disease were compared using chi‐square test. *p* < 0.05 is considered to be statistically significant.

Abbreviations: AD, Alzheimer's disease; ADL, activities of daily living; APOE, apolipoprotein E; BMI, body mass index; CDR, Clinical Dementia Rating; CDR‐SB, Clinical Dementia Rating Sum of Boxes; CN, cognitively normal; CTRP, C1q/tumor necrosis factor‐related protein; HbA1c, hemoglobin A1c; HDL‐C, high‐density lipoprotein cholesterol; IQR, interquartile range; LDL‐C, low‐density lipoprotein cholesterol; MMSE: Mini‐Mental State Examination; MoCA, Montreal Cognitive Assessment; NfL, neurofilament light chain; pT, tau phosphorylated at threonine; SD, standard deviation; TC, total cholesterol; TG, triglyceride.

Compared with the CN group, the proportion of *APOE4*‐positive (17.14 vs. 42.34, *p* < 0.0001) was increased in the AD group, while years of education (9 (8–12) vs. 6 (5.5–9), *p* < 0.0001), BMI (23.86 ± 3.52 vs. 21.35 ± 3.15, *p* < 0.0001), TG (1.78 ± 1.36 vs. 1.30 ± 0.51, *p* = 0.0106), and HDL‐C (1.63 ± 0.47 vs. 1.42 ± 0.41, *p* < 0.0001) were reduced (Table 1). There was no statistically significant difference between the two groups in age, sex, cigarette smoking, alcohol consumption, diabetes mellitus, hypertension, hyperlipidemia, coronary heart disease, fasting blood glucose, HbA1c, TC, and LDL‐C (each *p* > 0.05, Table 1).

By comparison with the CN group, MMSE score (28 (27–29) vs. 13 (9–19), *p* < 0.0001) and MoCA score (28 (26–29) vs. 8 (4–13), *p* < 0.0001) were reduced in the AD group, while ADL score (16 (16–16) vs. 35 (25–48), *p* < 0.0001) and CDR‐SB score (0 (0–0) vs. 12 (7–16), *p* < 0.0001) were increased (Table 1). Besides, plasma levels of pT217 (181.54 ± 73.19 vs. 239.15 ± 120.84, *p* = 0.0003), pT181 (1.69 ± 0.72 vs. 2.34 ± 1.04, *p* < 0.0001), and NfL (657.97 ± 282.27 vs. 839.04 ± 371.09, *p* < 0.0001) in the AD group were elevated (Table 1 and Figure S2).

Compared with the CN group, plasma levels of CTRP3 (102.84 ± 12.60 vs. 122.70 ± 15.60, *p* < 0.0001), CTRP4 (267.30 ± 23.35 vs. 335.42 ± 24.49, *p* < 0.0001), and CTRP14 (17.43 ± 3.31 vs. 19.27 ± 3.90, *p* < 0.0001) in the AD group were increased (Table 1 and Figure S2). Correlations between any two of the three CTRPs members were statistically significant among all subjects (Figure S3).

### 3.2 Association between plasma CTRPs with the presence of AD

Using the univariate binary logistic regression analysis, the years of education, *APOE4* status, BMI, TG, and HDL‐C at baseline were found to be correlated with the presence of AD (each *p* < 0.05) (Table S1). Furthermore, a multivariate logistic regression analysis adjusting these five variables and general risk factors for AD (i.e., age and sex) showed that plasma levels of CTRP3 (OR = 1.119, 95% CI: 1.082–1.158, *p* < 0.001), CTRP4 (OR = 1.230, 95% CI: 1.133–1.335, *p* < 0.001), and CTRP14 (OR = 1.158, 95% CI: 1.055–1.271, *p* = 0.002) were associated with the presence of AD (Table 2). Different combinations of CTRPs were also related to the presence of AD (Table 2).

**TABLE 2:** Independent factors associated with the presence of AD in the multivariate binary logistic regression analysis.

Model	Variable	OR value (95% CI)	*p* Value
Model 1	*APOE4*‐positive	3.685 (1.705–7.963)	0.001
	BMI	0.836 (0.753–0.929)	0.001
	TG	0.515 (0.324–0.821)	0.005
	CTRP3	1.119 (1.082–1.158)	<0.001
Model 2	Years of education	0.737 (0.603–0.900)	0.003
	*APOE4*‐positive	4.302 (1.028–18.002)	0.046
	BMI	0.770 (0.633–0.937)	0.009
	CTRP4	1.230 (1.133–1.335)	<0.001
Model 3	Years of education	0.897 (0.826–0.974)	0.009
	*APOE4*‐positive	3.314 (1.704–6.442)	<0.001
	BMI	0.819 (0.744–0.901)	<0.001
	HDL‐C	0.298 (0.144–0.618)	0.001
	TG	0.479 (0.315–0.728)	0.001
	CTRP14	1.158 (1.055–1.271)	0.002
Model 4	Years of education	0.648 (0.459–0.915)	0.014
	*APOE4*‐positive	13.949 (1.332–146.115)	0.028
	BMI	0.668 (0.497–0.896)	0.007
	CTRP3	1.168 (1.070–1.275)	0.001
	CTRP4	1.334 (1.142–1.556)	<0.001
Model 5	*APOE4*‐positive	3.261 (1.429–7.442)	0.005
	BMI	0.830 (0.743–0.927)	0.001
	TG	0.503 (0.308–0.821)	0.006
	CTRP3	1.131 (1.090–1.173)	<0.001
	CTRP14	1.243 (1.110–1.392)	<0.001
Model 6	Years of education	0.746 (0.605–0.920)	0.006
	BMI	0.743 (0.595–0.929)	0.009
	CTRP4	1.260 (1.141–1.391)	<0.001
	CTRP14	1.313 (1.059–1.626)	0.013
Model 7	Years of education	0.608 (0.396–0.933)	0.023
	*APOE4*‐positive	28.802 (1.428–580.942)	0.028
	BMI	0.546 (0.361–0.826)	0.004
	CTRP3	1.247 (1.085–1.433)	0.002
	CTRP4	1.505 (1.183–1.916)	0.001
	CTRP14	1.771 (1.160–2.702)	0.008

*Note*: Model 1: Age, Sex, Years of education, *APOE4*‐positive, BMI, HDL‐C, TG, and CTRP3. Model 2: Age, Sex, Years of education, *APOE4*‐positive, BMI, HDL‐C, TG, and CTRP4. Model 3: Age, Sex, Years of education, *APOE4*‐positive, BMI, HDL‐C, TG, and CTRP14. Model 4: Age, Sex, Years of education, *APOE4*‐positive, BMI, HDL‐C, TG, CTRP3, and CTRP4. Model 5: Age, Sex, Years of education, *APOE4*‐positive, BMI, HDL‐C, TG, CTRP3, and CTRP14. Model 6: Age, Sex, Years of education, *APOE4*‐positive, BMI, HDL‐C, TG, CTRP4, and CTRP14. Model 7: Age, Sex, Years of education, *APOE4*‐positive, BMI, HDL‐C, TG, CTRP3, CTRP4, and CTRP14. *p* < 0.05 is considered to be statistically significant.

Abbreviations: APOE, apolipoprotein E; BMI, body mass index; CI, confidence interval; CTRP, C1q/ tumor necrosis factor‐related protein; HDL‐C, high‐density lipoprotein cholesterol; OR, odds ratio; TG, triglyceride.

### 3.3 Correlations between plasma CTRPs with the clinical characteristics of AD

**FIGURE 1:**
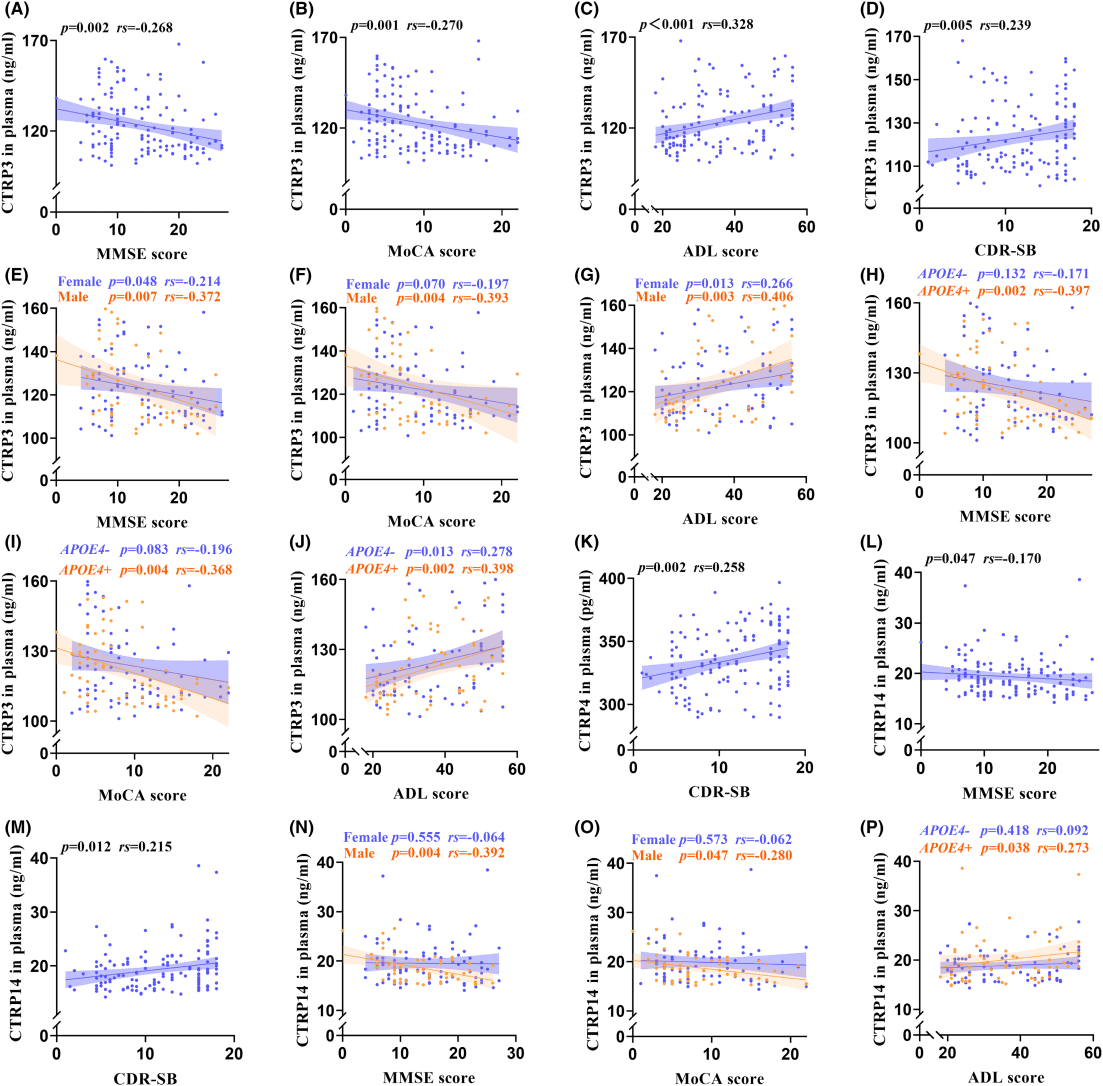
Correlations between plasma CTRPs with cognitive function, daily living abilities, and severity of dementia in AD patients. Correlations between plasma CTRP3 with scores of MMSE (A), MoCA (B), ADL (C), and CDR‐SB (D) in AD group. Associations between plasma CTRP3 with scores of MMSE, MoCA, and ADL in sex‐based subgroup (E‐G) and *APOE4‐*positive‐based subgroup (H–J). Relationships among plasma CTRP4 with CDR‐SB score (K), plasma CTRP14 with MMSE score (L), and plasma CTRP14 with CDR‐SB score (M). Relationships between plasma CTRP14 with MMSE score (N) and MoCA score (O) in male AD patients. The link between plasma CTRP14 with ADL score in *APOE4‐*positive AD patients (P). AD, Alzheimer's disease; ADL, activities of daily living; APOE, apolipoprotein E; CDR‐SB, Clinical Dementia Rating Sum of Boxes; CTRP, C1q/tumor necrosis factor‐related protein; MMSE, Mini‐Mental State Examination; MoCA, Montreal Cognitive Assessment. Correlation analyses were performed using Spearman's rank correlation or Pearson's correlation. *p* < 0.05 is considered to be statistically significant.

In the AD group, the plasma CTRP3 was negatively correlated with MMSE score (*rs* = −0.268, *p* = 0.002) and MoCA score (*rs* = −0.270, *p* = 0.001), while positively correlated with ADL score (*rs* = 0.328, *p* < 0.001) and CDR‐SB score (*rs* = 0.239, *p* = 0.005) (Figure 1A–D). Likewise, most of these correlations were also statistically significant in sex‐ or *APOE4*‐based subgroups (Figure 1E‐J). CTRP4 only had a positive correlation with CDR‐SB score in the AD group (*rs* = 0.258, *p* = 0.002) (Figure 1K). CTRP14 showed marked correlations with MMSE score (*rs* = −0.170, *p* = 0.047) and CDR‐SB score (*rs* = 0.215, *p* = 0.012) (Figure 1L,M), with male AD patients taking negative correlations of CTRP14 with MMSE score (*rs* = −0.392, *p* = 0.004) and MoCA score (*rs* = −0.280, *p* = 0.047) (Figure 1N,O), while *APOE4*‐positive AD patients having a positive correlation of CTRP14 with ADL score (*rs* = 0.273, *p* = 0.038) (Figure 1P). Other correlations were not of statistical significance (each *p* > 0.05) (Figure S4). In addition, most of the correlations between CTRPs and scores of MMSE, MoCA, and ADL showed statistical significance in all subjects, sex‐based subgroups, and *APOE4*‐based subgroups (Figure S5).

### 3.4 Correlations between plasma CTRPs with representative blood biomarkers of AD

**FIGURE 2:**
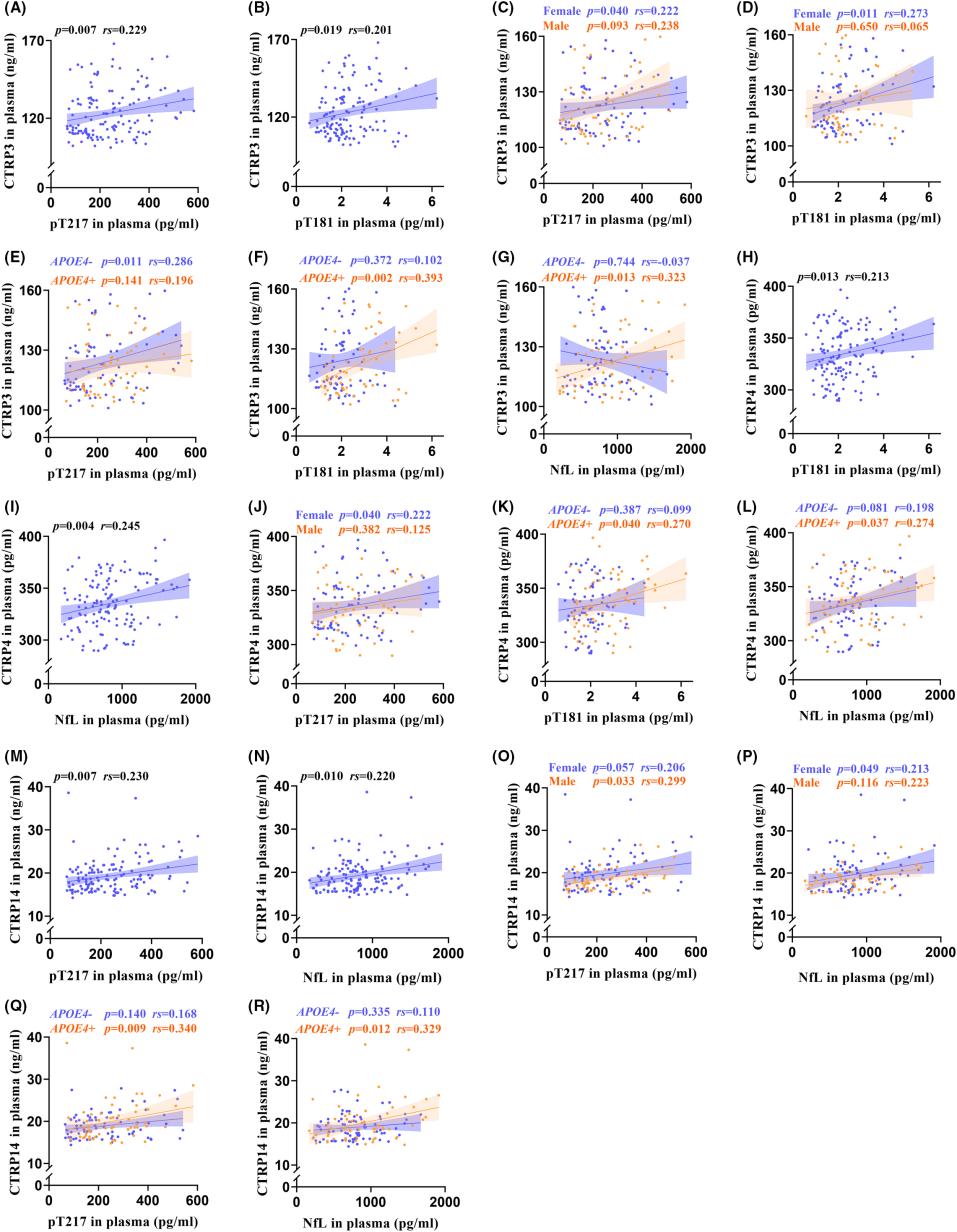
Correlations between plasma CTRPs with representative blood biomarkers in AD patients. Correlations among plasma CTRP3 with pT217 (A) and pT181 (B), plasma CTRP4 with pT181 (H) and NfL (I), and plasma CTRP14 with pT217 (M) and NfL (N) in AD group. Associations between plasma CTRP3 with pT217 and pT181 in sex‐based subgroup (C and D) and in *APOE4‐*positive‐based subgroup (E and F). Relationships between plasma CTRP3 with NfL (G), plasma CTRP4 with pT181 (K), and plasma CTRP4 with NfL (L) in *APOE4*‐positive AD patients. The link between plasma CTRP4 with pT217 in female AD patients (J). Relationships between plasma CTRP14 with pT217 and NfL in sex‐based subgroup (O and P) and *APOE4‐*positive‐based subgroup (Q and R). AD, Alzheimer's disease; APOE, apolipoprotein E; CTRP, C1q/tumor necrosis factor‐related protein; NfL, neurofilament light chain; pT, tau phosphorylated at threonine. Correlation analyses were performed using Pearson's correlation or Spearman's rank correlation. *p* < 0.05 is considered to be statistically significant.

In the AD group, the plasma CTRP3 showed significant positive correlations with plasma pT217 (*rs* = 0.229, *p* = 0.007) and pT181 (*rs* = 0.201, *p* = 0.019) (Figure 2A,B). Likewise, most of these correlations were also statistically significant in sex‐ or *APOE4*‐based subgroups (Figure 2C–G). The plasma CTRP4 showed positive correlations with plasma pT181 (*rs* = 0.213, *p* = 0.013) and NfL (*r* = 0.245, *p* = 0.004) in the AD group (Figure 2H,I). It was also of statistical significance in CTRP4's correlations with pT217 (*rs* = 0.222, *p* = 0.040) in female AD patients, and pT181 (*rs* = 0.270, *p* = 0.040) and NfL (*rs* = 0.274, *p* = 0.037) in *APOE4*‐positive AD patients (Figure 2J–L). The plasma CTRP14 had positive correlations with plasma pT217 (*rs* = 0.230, *p* = 0.007) and NfL (*rs* = 0.220, *p* = 0.010) in AD patients (Figure 2M,N). Besides, majority of these correlations were also statistically significant in sex‐ or *APOE4*‐based subgroups (Figure 2O–R). However, other correlations were not statistically significant in AD patients (Figure S6). Besides, most of the correlations between plasma CTRPs with pT217, pT181, and NfL in all subjects as well as sex‐ or *APOE4*‐based subgroups showed statistical significance (Figure S7).

### 3.5 Diagnostic power of plasma CTRPs for AD

**FIGURE 3:**
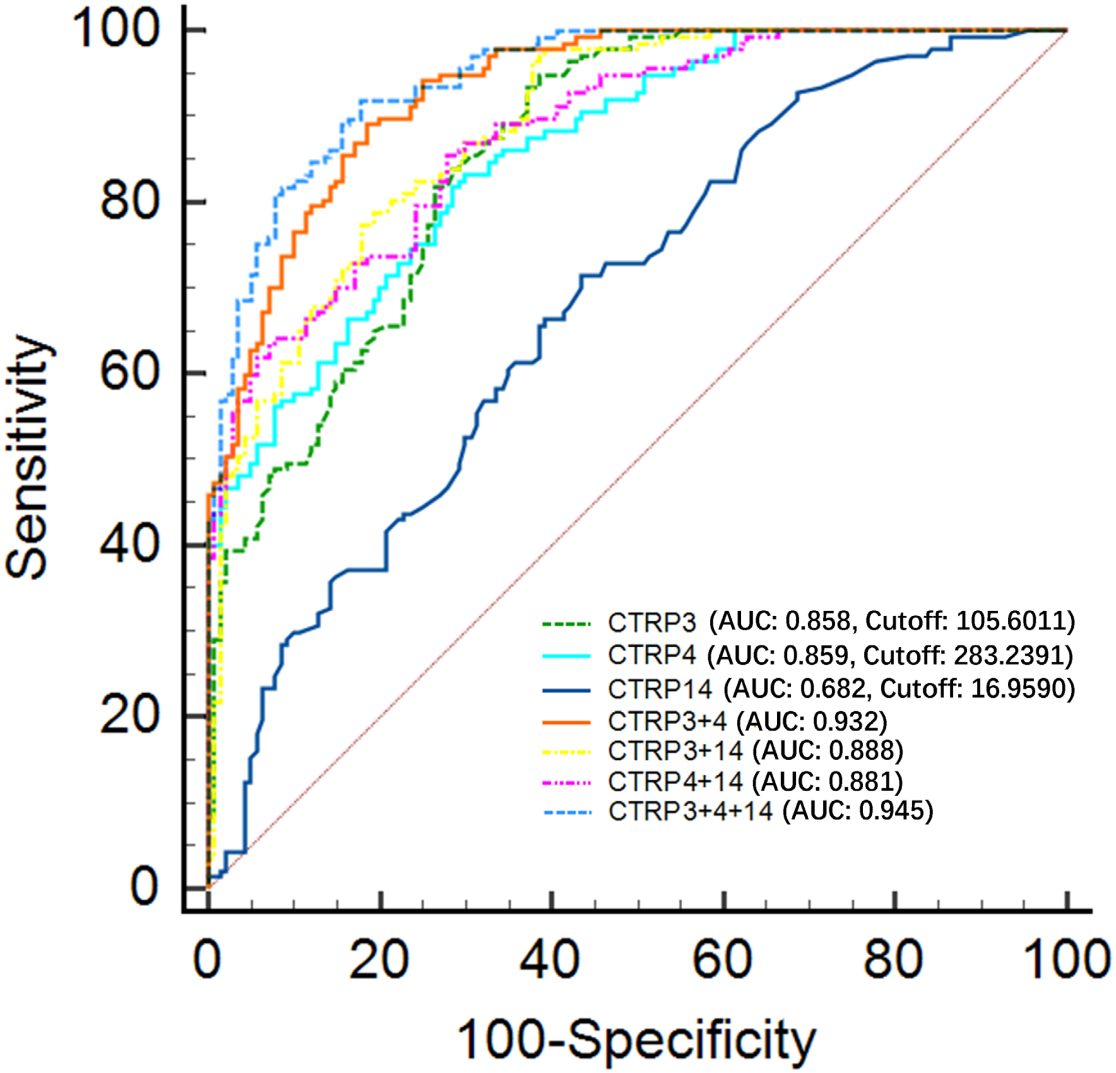
Diagnostic power of plasma CTRPs for AD. ROC analyses of CTRP3, 4, 14, and their combinations. AD, Alzheimer's disease; AUC, area under the curve; CTRP, C1q/tumor necrosis factor‐related protein; ROC, receiver operating characteristic. Cutoff values were calculated using Youden's Index. *p* < 0.05 is considered to be statistically significant.

To analyze the performance of plasma CTRPs and their combinations to distinguish AD patients from CN controls, a ROC analysis was conducted. Each indicator featured significantly high AUC, which far exceeded the random chance (AUC of 50%) (each *p <* 0.001, AUCs ranged from 0.682 to 0.945) with the cutoff values of CTRP3 (105.6011), CTRP4 (283.2391), and CTRP14 (16.9590) (Figure 3). DeLong's test was used to compare AUCs of these indicators mutually (Table S2). Both AUCs of CTRP3 (*Z* = 4.353, *p* < 0.0001) and CTRP4 (*Z* = 4.549, *p* < 0.0001) were higher than the AUC of CTRP14 (Table S2).

To determine whether CTRPs increased the diagnostic efficacy of conventional factors for AD, all aforementioned variables (age, sex, years of education, *APOE4*‐positive, BMI, TG, and HDL‐C) were used to establish the basic model for AD diagnosis, namely Model 0 where AUC of the ROC curve was greater than the random probability AUC (AUC = 0.825, *p* < 0.001). Then, CTRP3, 4, 14, and their combinations were added to Model 0, namely Models 1–7, respectively. AUCs of Models 1–7 were greater than the random probability AUC (each *p* < 0.001, with an AUC range of 0.838–0.962) (Figure S8). Furthermore, DeLong's test showed that except for Model 3, the diagnostic power of the other six models was higher than Model 0 (each *p* < 0.0001) (Table 3).

**TABLE 3:** Pairwise comparison of the area under receiver operator characteristic curves by DeLong's test.

	Difference between areas	*Z* statistic	*p*‐Value
Model 0 vs. Model 1	0.082	4.334	<0.0001
Model 0 vs. Model 2	0.090	4.553	<0.0001
Model 0 vs. Model 3	0.013	1.346	0.1784
Model 0 vs. Model 4	0.129	5.879	<0.0001
Model 0 vs. Model 5	0.101	4.890	<0.0001
Model 0 vs. Model 6	0.100	4.893	<0.0001
Model 0 vs. Model 7	0.137	6.039	<0.0001

*Note*: Model 0: Age, Sex, Years of education, *APOE4*‐positive, BMI, TG, and HDL‐C. Model 1: Age, Sex, Years of education, *APOE4*‐positive, BMI, TG, HDL‐C, and CTRP3. Model 2: Age, Sex, Years of education, *APOE4*‐positive, BMI, TG, HDL‐C, and CTRP4. Model 3: Age, Sex, Years of education, *APOE4*‐positive, BMI, TG, HDL‐C, and CTRP14. Model 4: Age, Sex, Years of education, *APOE4*‐positive, BMI, TG, HDL‐C, CTRP3, and CTRP4. Model 5: Age, Sex, Years of education, *APOE4*‐positive, BMI, TG, HDL‐C, CTRP3, and CTRP14. Model 6: Age, Sex, Years of education, *APOE4*‐positive, BMI, TG, HDL‐C, CTRP4, and CTRP14. Model 7: Age, Sex, Years of education, *APOE4*‐positive, BMI, TG, HDL‐C, CTRP3, CTRP4, and CTRP14. *p* < 0.05 is considered statistically significant.

Abbreviations: APOE, apolipoprotein E; BMI, body mass index; CTRP, C1q/tumor necrosis factor‐related protein; HDL‐C, high‐density lipoprotein cholesterol; TG, triglyceride.

Finally, in order to demonstrate the model's generalization ability in the real world, an internal validation was conducted on 277 samples through bootstrap resampling for 1000 times. It showed that the model's validation efficiency and risk threshold were significantly higher than the random probability, and the calibration curve showed that the actual curve was close to the ideal curve reflected as the C‐index (CTRP3: 89.3%, CTRP4: 98.8%, and CTRP14: 81.9%), indicating that the deviation between the models' predicted results and the actual results was small (CTRP3 CI: 95%, mean absolute error (MAE) = 0.019, CTRP4 CI: 95%, MAE = 0.012, CTRP14 CI: 95%, MAE = 0.021), suggesting that the model in this study had a good effect (Figure S9).

## 4 DISCUSSION

Previously, we proposed a new concept of the brain–visceral adipose tissue axis and sorted out its potential involvement in the pathogenesis of AD, in which a key mediator is adipokines produced by the adipose tissue.^6^ The adipose tissue is the main site producing and secreting CTRPs, and nominally traditional adipokines (e.g., leptin, resistin, adiponectin, chemerin, and lipocalin‐2) have been widely studied on their associations with clinical characteristics and pathogenesis of AD.^6^ In the present study, plasma levels of three CTRPs members were higher in the AD group than those in the CN group and were remarkably associated with the presence of AD, suggesting that CTRPs, as a newly discovered adipokine family, are also to be associated with AD.

Cognitive impairment and decreased abilities of daily living are core clinical characteristics of AD, so we employed MoCA and MMSE, ADL, and CDR‐SB for the assessment of cognitive function, living status, and severity of dementia, respectively. In the present study, three CTRP members showed varying degrees of correlations with these four indicators of scores in the AD group, with CTRP3 performing more comprehensively. Likewise, we adopted pT181 and pT217 to evaluate the hyperphosphorylation of tau protein, and NfL to examine the degree of neurodegeneration. Each CTRP member showed correlations with a majority of these representative biomarkers in the AD group. It should be pointed out that both female sex and *APOE4* represent the strongest risk factors for AD in addition to aging, even though there is currently no consensus on which one is stronger^25^, ^26^, ^27^; thus, the similar correlations in the subgroups based on sex or *ApoE4* status were further investigated following previous studies.^19^, ^28^ All correlation analysis results indicate that CTRPs are associated with the symptomatic severity and neurodegeneration degree of AD.

As the molecular structural characteristic of the CTRPs family, the complement protein C1q represents the first subcomponent of the C1 complex which binds to various ligands and serves as a recognition target for several classical signal cascades.^29^ In detail, both CTRP3 and CTRP14 have four different domains including a signal peptide at the N‐terminus, a collagenous domain with various lengths of Gly‐X‐Y repeats, a short variable region, and a globular C‐terminal domain homologous to C1q,^30^ while CTRP4 has two C‐terminal globular domains connected by a short linker and lacks the collagen domain.^30^ CTRPs have multiple biological regulatory functions on immunity, metabolism, and inflammation, supporting their crucial role in a variety of diseases such as obesity, cardiovascular disease, and tumors.^13^ All the findings in the present study have preliminarily revealed the association between CTRPs and AD at the phenotypic level. It must be pointed out that although the diagnosis of “probable AD” under the NINCDS‐ADRDA criteria is reliable,^31^ multiple studies have confirmed that the criteria have approximately a sensitivity of 80% and a specificity of 70% using pathological‐confirmed AD as a reference.^32^ To some extent, this limited the reliability of our further inferences about the involvement of CTRPs in the pathogenesis of AD from the findings of this study. Thus, we conducted the following literature review and theoretical analysis.

CTRP3 is permeable to the BBB not requiring the involvement of lipids and glucose in vivo, and then exerts biological effects through various signaling pathways.^9^ The overexpression of CTRP3 attenuated neuronal apoptosis, neuroinflammation, brain damage, and cognitive dysfunction by regulating AMP‐activated protein kinase (AMPK)/sirtuin 1 (SIRT 1) and phosphoinositide 3‐kinase (PI3K)/AKT pathways in aged rats anesthetized with isoflurane.^10^ Lacking CTRP3 exacerbated depression‐type behaviors (e.g., decreased sucrose consumption and locomotor activity), neuronal death, expression of cleaved caspase‐3, and pro‐inflammatory responses by inhibiting p38 and Jun N‐terminal kinase (JNK)/mitogen‐activated protein kinase (MAPK) pathways in a depression mouse model.^33^ The overexpression of CTRP3 attenuated oxidative stress, enhanced mitochondrial biogenesis, and induced changes in a series of regulatory molecules such as BCL‐2 expression, BAX expression, and Caspase‐3 activity via both AMPK/nuclear factor erythroid 2‐related factor 2 (NRF2)/antioxidant response element (ARE) and AMPK/SIRT1/peroxisome proliferator‐activated receptor gamma coactivator‐1α (PGC‐1α) pathways in oxygen–glucose deprivation/reperfusion‐induced hippocampal neurons.^34^, ^35^ Administration of CTRP3 reduced reactive oxygen species and malondialdehyde production, increased the level of glutathione, attenuated cerebral edema and BBB disruption, promoted angiogenesis, and improved neurological defects via both protein kinase A (PKA) and AMPK/hypoxia inducing factor‐1α (HIF‐1α)/vascular endothelial growth factor (VEGF) pathways in an intracerebral hemorrhage rat model.^36^, ^37^ CTRP3 inhibited myelin oligodendrocyte glycoprotein‐induced interleukin (IL) ‐17 production in T cells and dendritic cells by activating adiponectin receptor 2.^38^

In divergent vertebrates such as humans, mice, and zebrafish, the expression of CTRP4 was predominantly detected in adipose tissue and central nervous system (CNS).^13^, ^39^ In detail, CTRP4 is mainly expressed in spinal cord, midbrain, hindbrain, hypothalamus, cerebral cortex, and hippocampus in mice.^13^ Lacking of CTRP4 impaired associative learning and memory and changed the expression of activity‐regulated cytoskeleton‐associated protein (Arc), phosphodiesterase 4D, and c‐fos in the hippocampus and cortex in female mice.^13^ The overexpression of CTRP4 in the hypothalamus reduced TNF‐α and IL‐6 levels in both brain and blood and inhibited hypothalamic NF‐κB signaling and microglia activation in mice.^40^ During the ascending phase of herpes simplex encephalitis, the expression of CTRP4 in the brain was closely related to serum levels of IL‐6 and TNF‐α, volumes of brain damage, and the decline in MMSE scores.^41^ In addition, CTRP4 was found to play a central role in energy metabolism.^12^ Overnight fasting followed by feeding induced a hypothalamic expression of CTRP4.^12^ Intracerebral administration of CTRP4 inhibited food intake and altered overall energy balance by inhibiting the expression of hypothalamic appetite neuropeptides such as neuropeptide Y and agouti‐related neuropeptide.^12^

CTRP14 is encoded by homologous genes highly expressed in both brain and adipose tissue and is believed to be closely related to cognitive and synaptic function.^42^, ^43^ Lack of CTRP14 altered the ambulatory activity in a sex‐ and nutritional state‐dependent manner. The ambulatory activity was reduced in CTRP14 knock‐out male mice in ad libitum fed, fasted, and refed states; however, female CTRP14 knock‐out mice had higher total physical activities during both food deprivation and refeeding periods.^44^ A single nucleotide polymorphism of CTRP14 (rs962888) was found to be associated with cerebral white matter hyperintensities in patients with stroke.^45^ As one of the synaptic organizers, CTRP14 regulated the synaptic formation and maintenance of parallel and climbing fiber of Purkinje cells in the cerebellum.^16^ Restriction of the CTRP14 expression to climbing fibers in the cerebellum was necessary for their proper innervation of target Purkinje cells.^46^ Besides, CTRP14 promoted spinogenesis and parallel fibers synaptogenesis in Purkinje cells in a brain angiogenesis inhibitor‐3 (an adhesion G‐protein‐coupled receptor)‐dependent manner.^46^

To sum up, CTRPs are extremely closely related to neuropsychological activities such as cognition, depression, and behavioral abnormalities, and are widely involved in the regulation of neuroinflammation, apoptosis, and synaptic plasticity. Especially, many of the upstream and downstream signaling pathways attributed to CTRPs are also involved in the metabolism of Aβ or tau. Therefore, the previous literature on CTRPs above strongly suggests the potential involvement of CTRPs in the pathogenesis of AD.

The present study has several limitations as below. As a cross‐sectional study, it only uncovered associations between plasma CTRPs and AD but could not determine the causal relationship between them. Due to the fact that most patients' families were not willing to undergo lumbar puncture and positron emission tomography tests, the inclusion criteria for AD had to adopt the clinical criteria of NINCDS‐ADRDA rather than the biomarker‐based criteria of NIA‐AA. Besides, the Eighth People's Hospital of Chengdu is a chronic disease hospital and municipal sanatorium for cadres. Some of patients with AD were chronically hospitalized and had only undergone head computed tomography scanning. Thus, we did not determine the association between CTRPs and neuroimaging. Moreover, CTRP family has 15 members, but we only focused on CTRP3, 4, and 14 in the light of published literature, which may lead to the potential connection between other CTRPs and AD being ignored. In addition, it is most theoretically reasonable to use a set of samples independent of the exploratory study for external verification; however, we did not have any more independent samples. Thus, we had to resort to the resampling method for the internal validation referring to other studies.^47^, ^48^, ^49^

## 5 CONCLUSION

Taken together, the present study preliminarily uncovered that the plasma CTRPs were associated with the presence, clinical characteristics, and representative blood biomarkers of AD and had certain diagnostic power for AD. To our knowledge, this is the first report ever revealing associations between plasma CTRPs with AD so far. The related mechanism of CTRPs in the pathogenesis of AD is worthy of an in‐depth study in the future.

## AUTHOR CONTRIBUTIONS

SY, WL, and YX carried out the study conception and design. HX, JZ, TY, SG, LY, and FY performed the material preparation, biochemical and molecular investigation, and data collection. XH, QW, and JZ analyzed the data. XH, JZ, and YX wrote the first draft of the manuscript. All authors commented on the previous versions of the manuscript and read and approved the final manuscript.

## CONFLICT OF INTEREST STATEMENT

The authors declare that they have no conflict of interest.

## Supporting information


Figure S1

Figure S2

Figure S3

Figure S4

Figure S5

Figure S6

Figure S7

Figure S8

Figure S9

Table S1

Table S2

## Data Availability

The data from the current study are available from the corresponding author upon reasonable request.
