# Supplementary figures and images for: Association between plasma CTRPs with cognitive impairment and neurodegeneration of Alzheimer's disease
Source: CNS Neurosci Ther. 2024 Feb 9;30(2):e14606. doi: 10.1111/cns.14606 (PMC10853890; doi:10.1111/cns.14606)

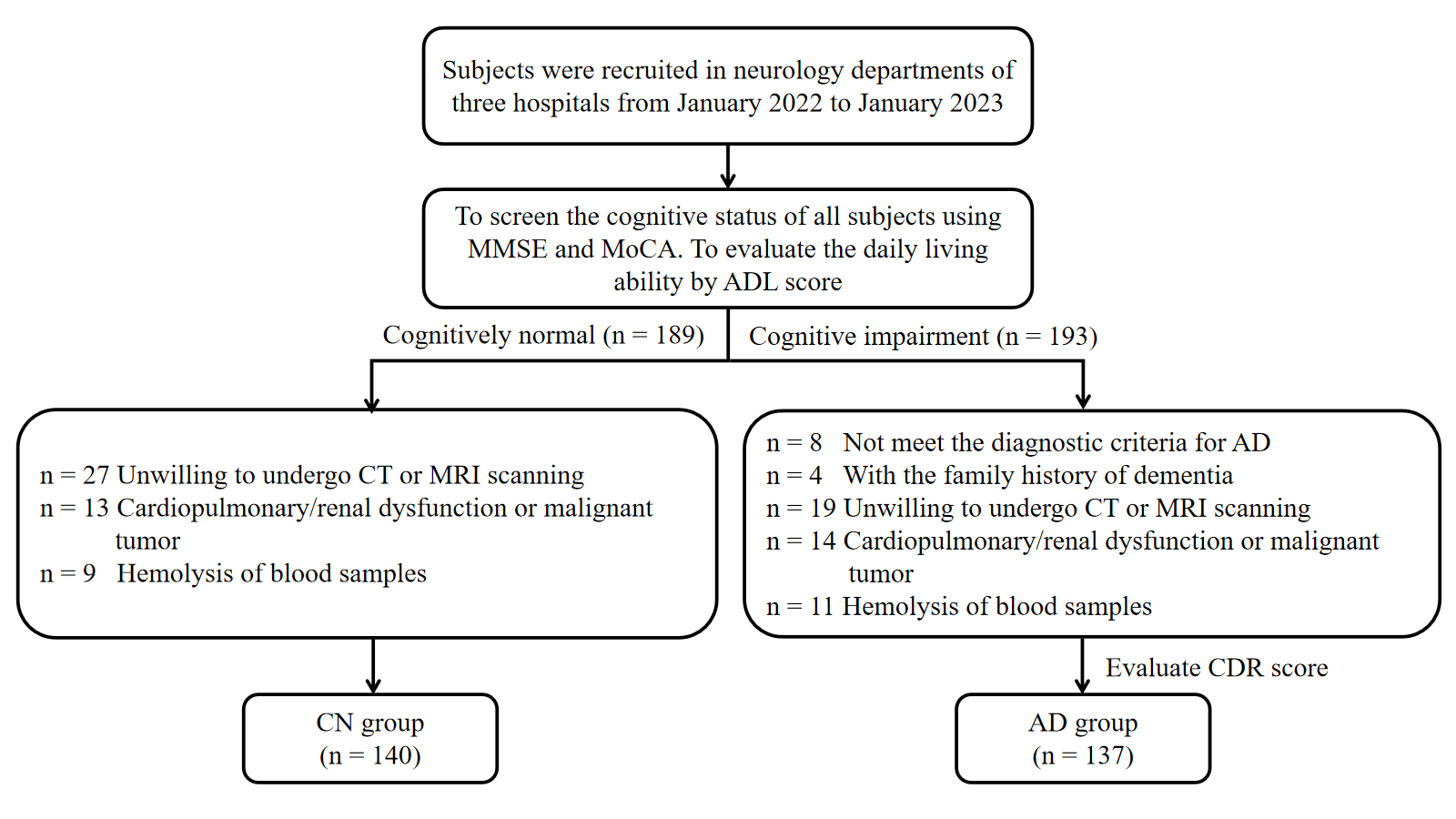

Supplement: Supplementary file 1 — Figure S1 [file CNS-30-e14606-s003.tif]

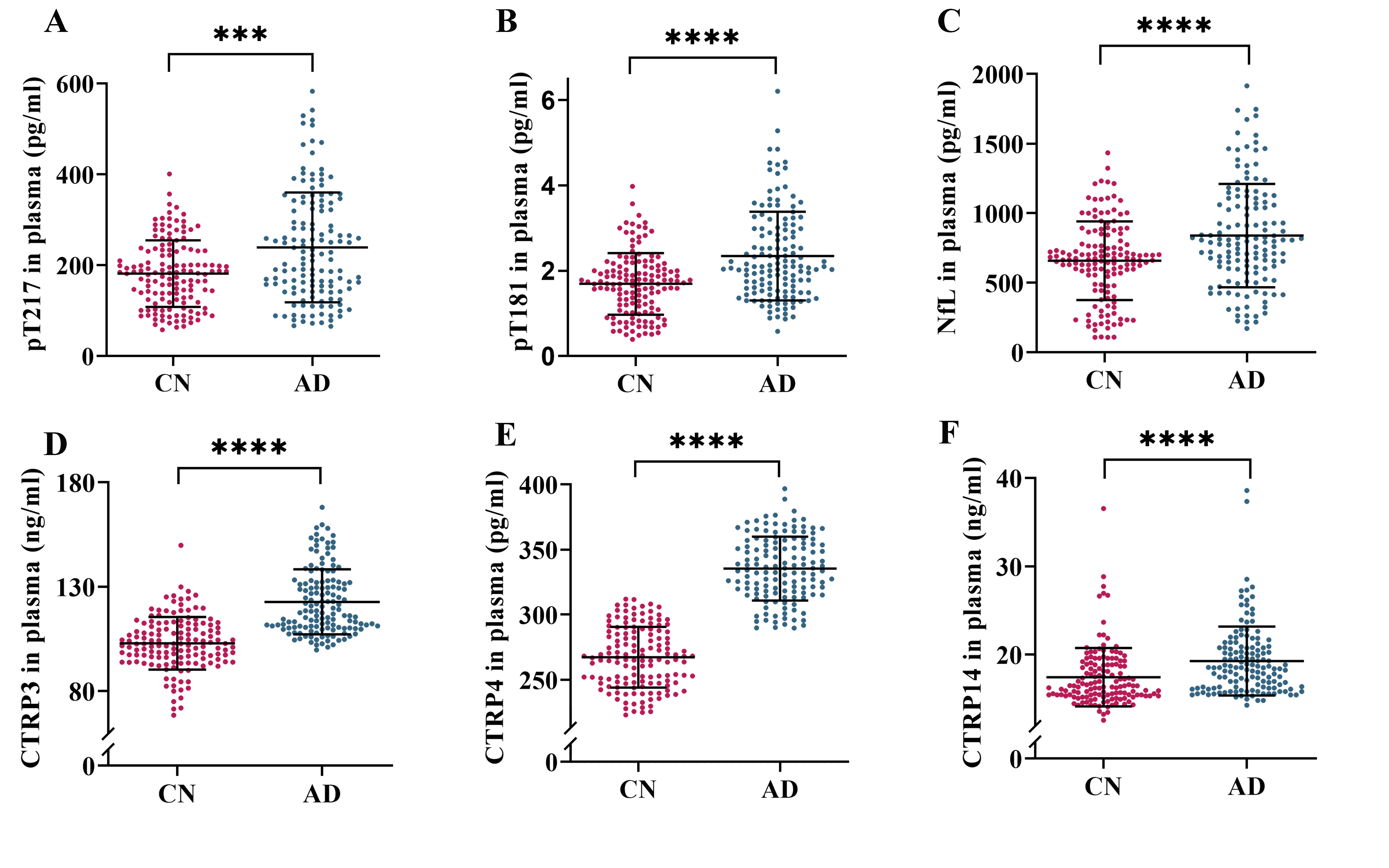

Supplement: Supplementary file 2 — Figure S2 [file CNS-30-e14606-s006.tif]

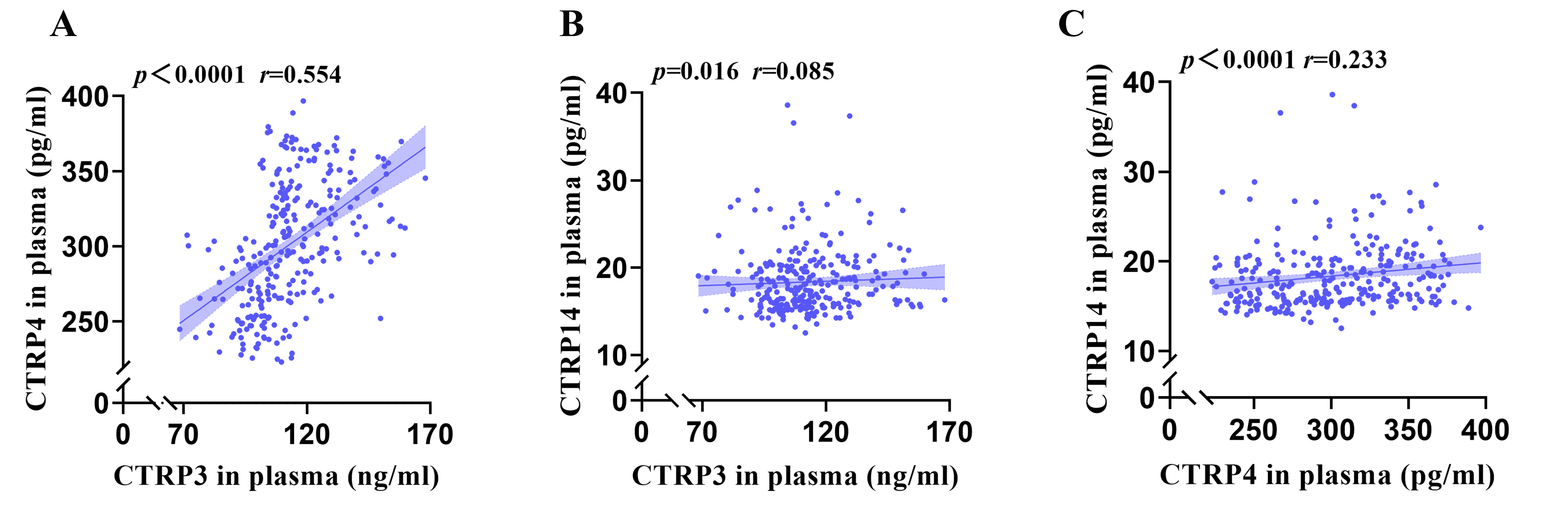

Supplement: Supplementary file 3 — Figure S3 [file CNS-30-e14606-s007.tif]

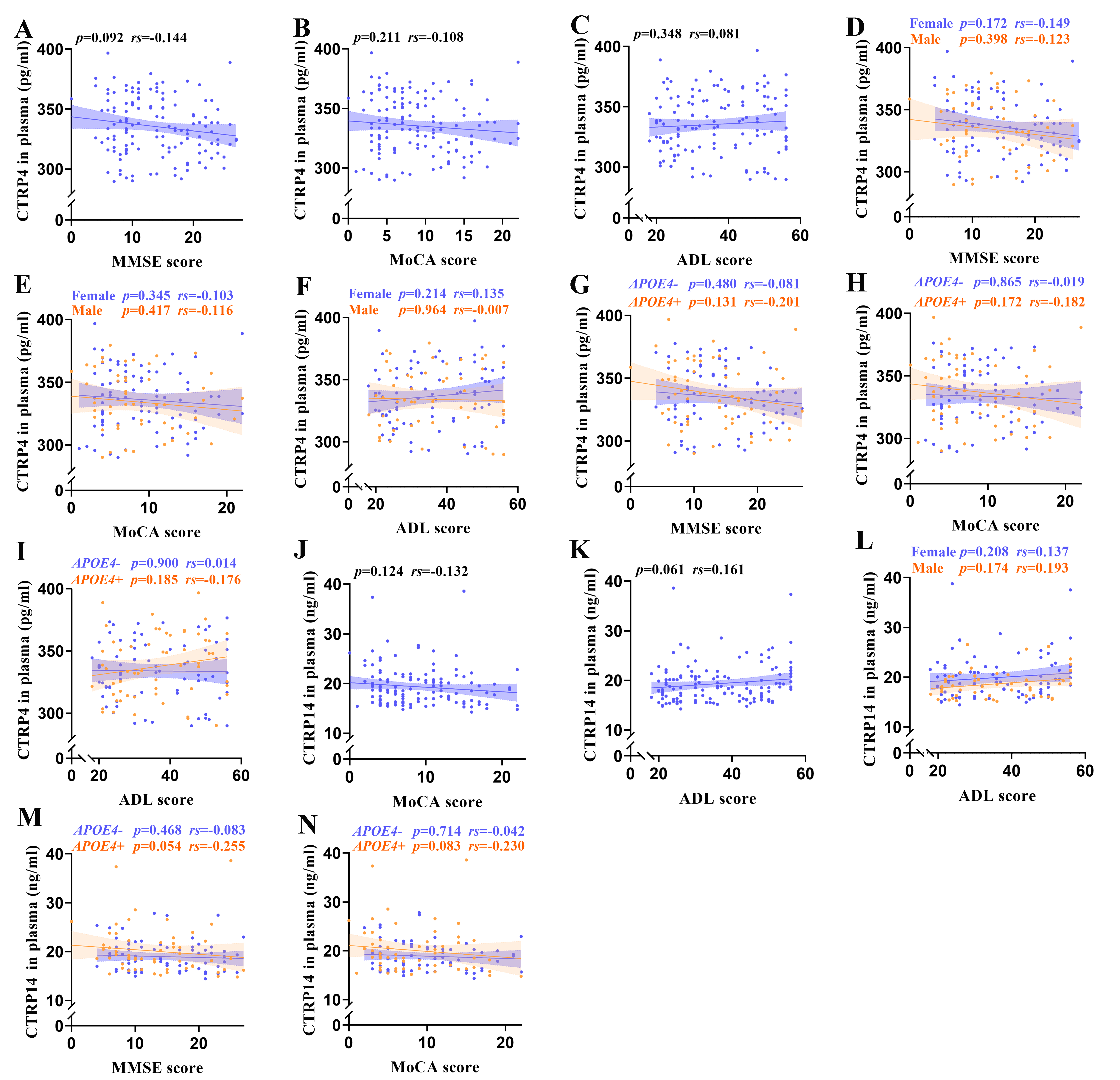

Supplement: Supplementary file 4 — Figure S4 [file CNS-30-e14606-s010.tif]

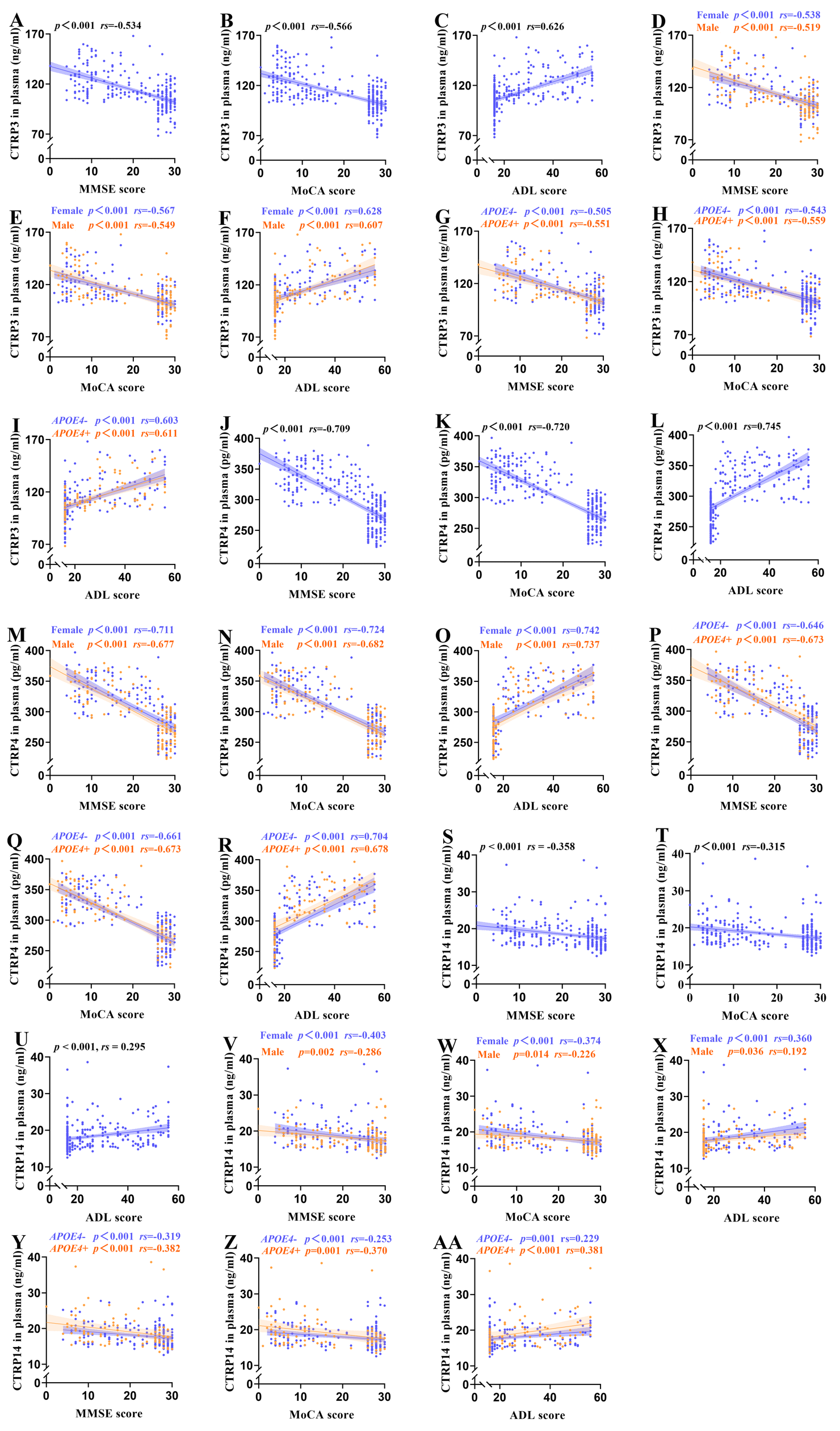

Supplement: Supplementary file 5 — Figure S5 [file CNS-30-e14606-s004.tif]

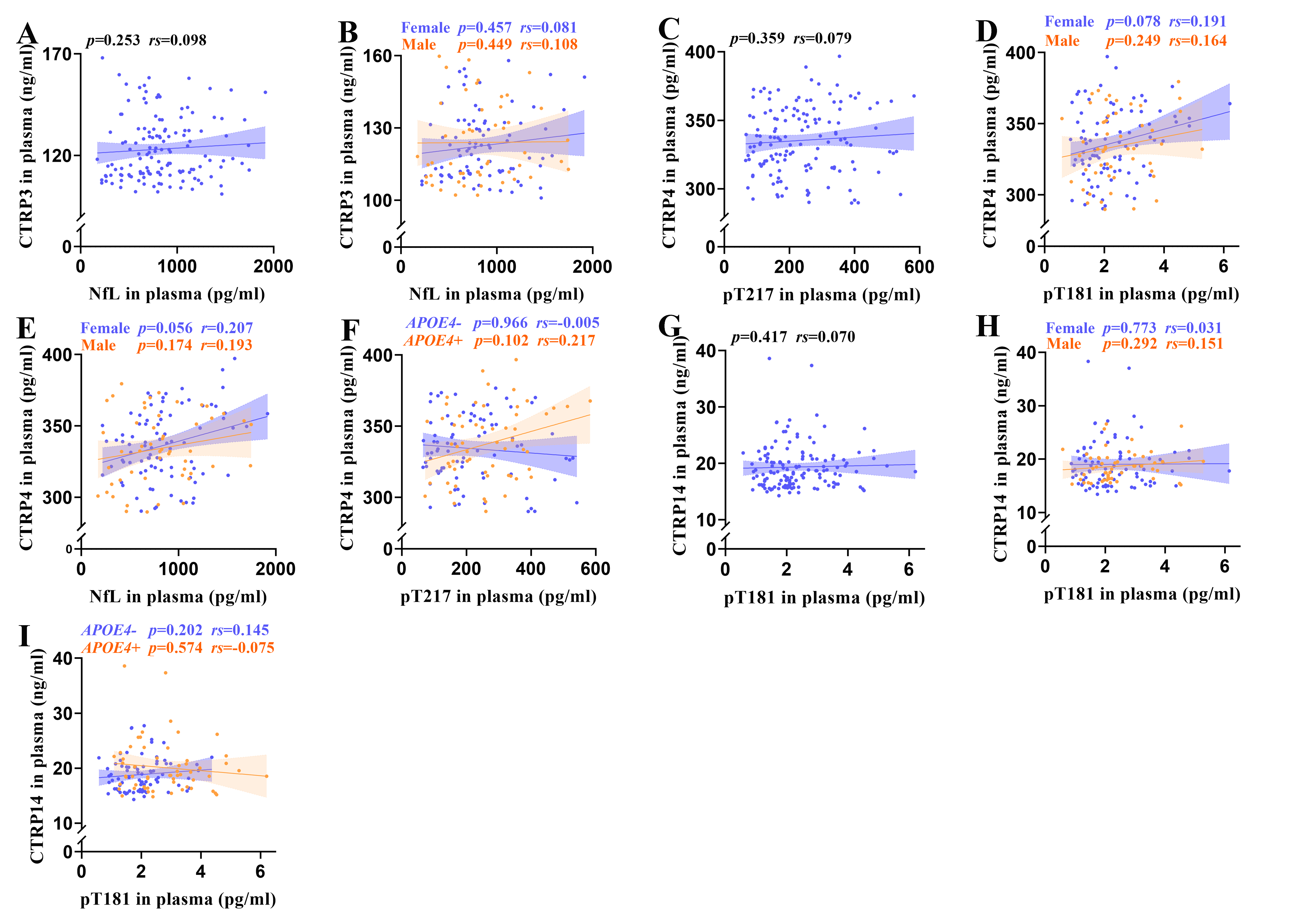

Supplement: Supplementary file 6 — Figure S6 [file CNS-30-e14606-s005.tif]

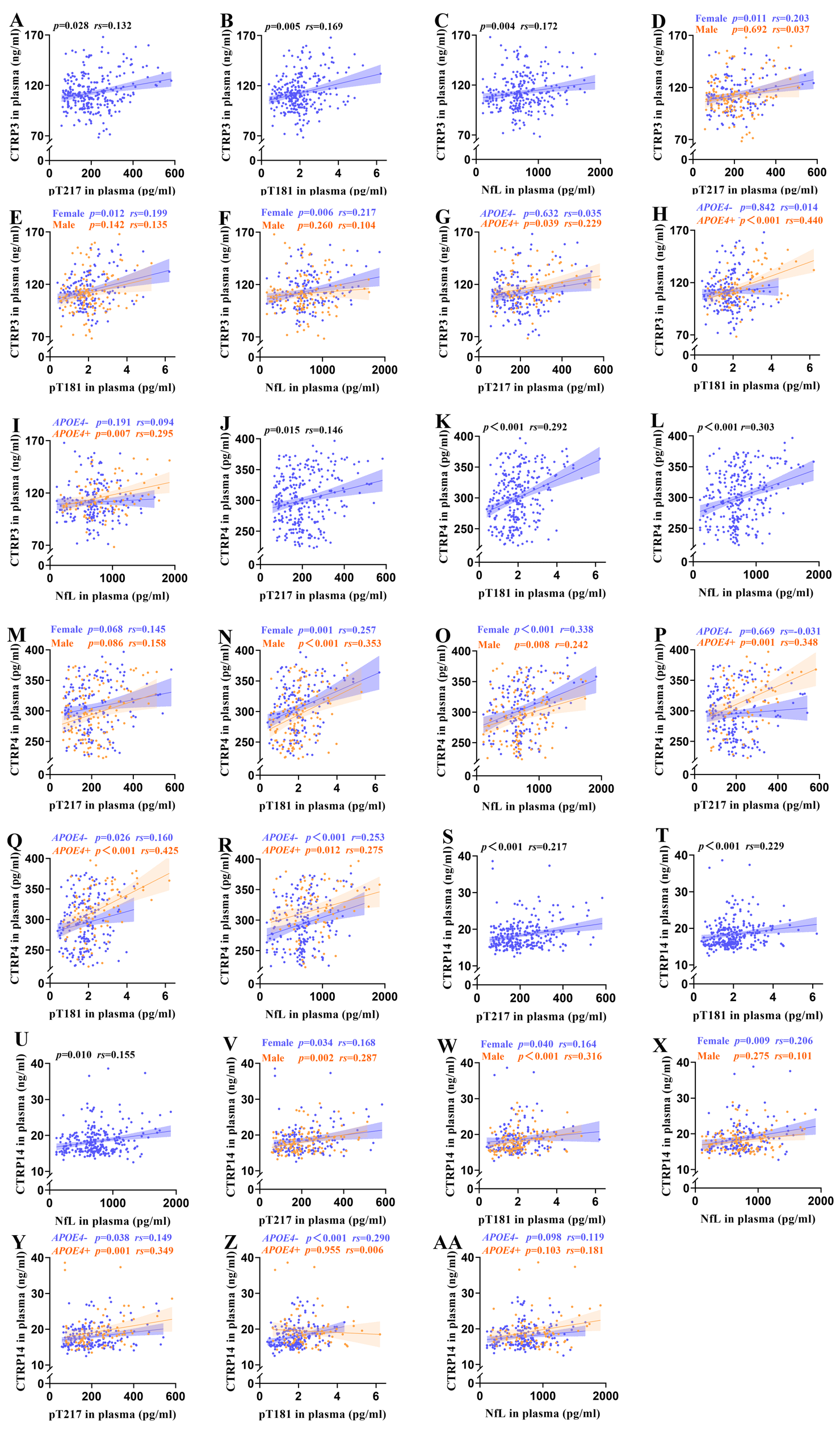

Supplement: Supplementary file 7 — Figure S7 [file CNS-30-e14606-s009.tif]

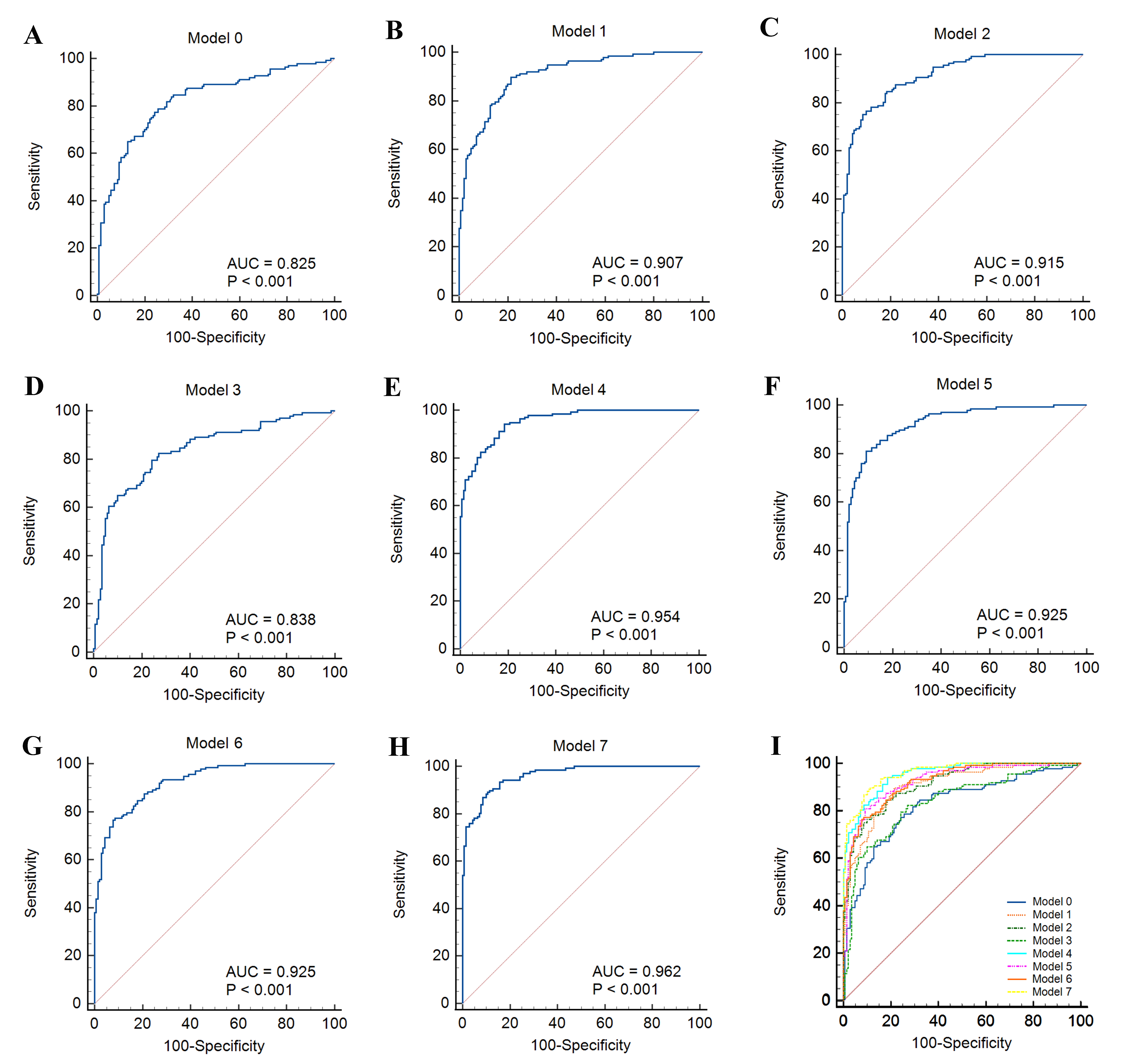

Supplement: Supplementary file 8 — Figure S8 [file CNS-30-e14606-s011.tif]

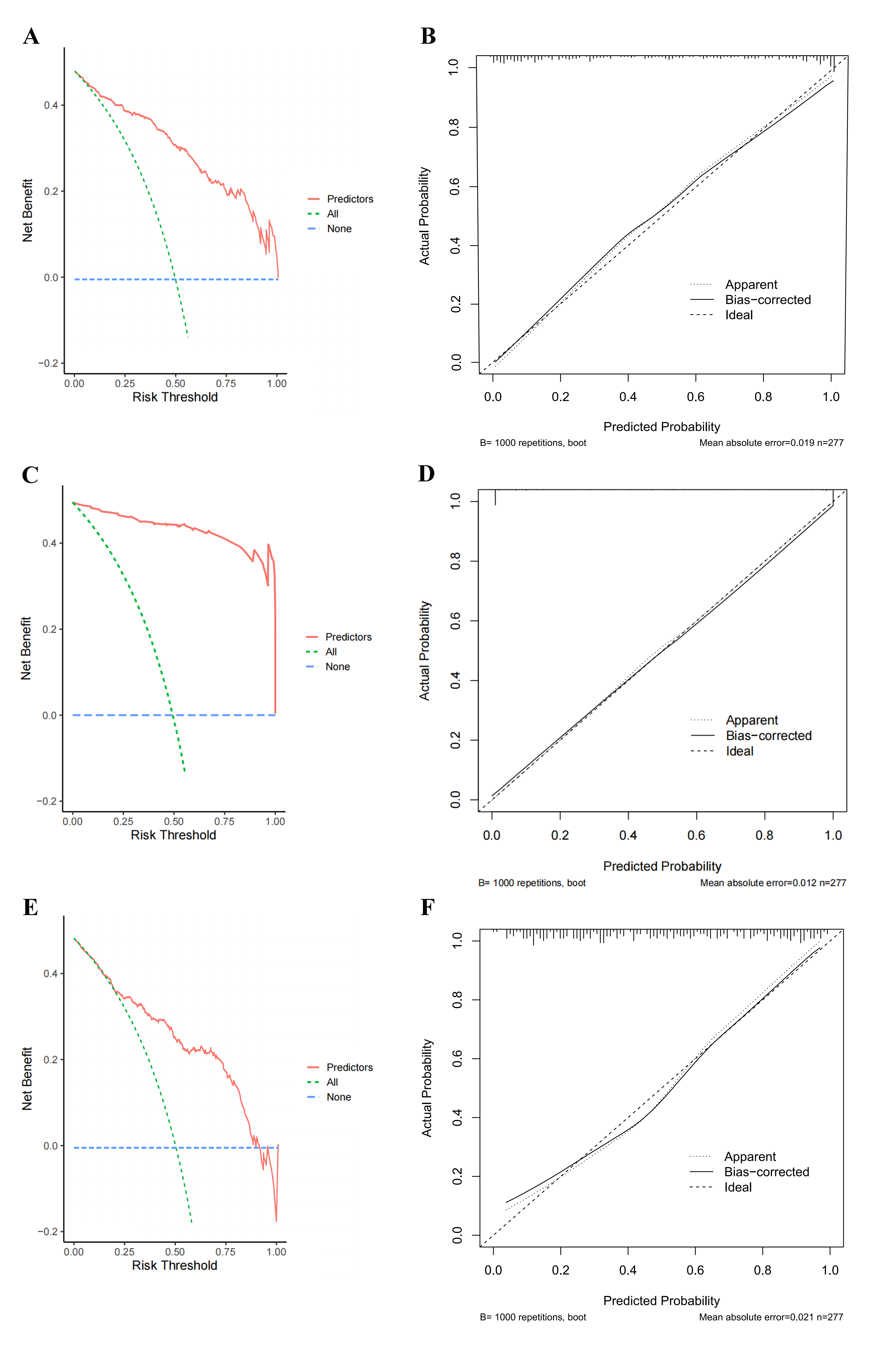

Supplement: Supplementary file 9 — Figure S9 [file CNS-30-e14606-s002.tif]
