# Supplementary material for: Association between plasma CTRPs with cognitive impairment and neurodegeneration of Alzheimer's disease
Source: CNS Neurosci Ther. 2024 Feb 9;30(2):e14606. doi: 10.1111/cns.14606 (PMC10853890; doi:10.1111/cns.14606)
Supplement: Supplementary file 10 — Table S1 [file CNS-30-e14606-s008.pdf]

Supplemental Table 1. Screening of risk factors associated with the presence of AD by the univariate binary logistic regression analysis

Variable	OR value (95% CI)	*p* value
Age (years)	1.019 (0.990- 1.049)	0.197
Sex (No.)	0.628 (0.389- 1.014)	0.057
Years of education (years)	0.889 (0.829-0.954)	0.001
APOE4-positive (No.)	3.549 (2.037-6.181)	< 0.001
BMI (kg/m^2)	0.786 (0.723-0.855)	< 0.001
Cigarette smoking (No.)	1.074 (0.600- 1.924)	0.810
Alcohol consumption (No.)	1.077 (0.587- 1.979)	0.810
Diabetes mellitus (No.)	1.399 (0.811-2.413)	0.227
Hypertension (No.)	1.314 (0.818-2.110)	0.258
Hyperlipidemia (No.)	0.673 (0.419- 1.082)	0.102
Coronary heart disease (No.)	0.986 (0.558- 1.740)	0.961
Fasting blood glucose (mmol/L)	0.880 (0.740- 1.047)	0.148
HbA1c (mg/dL)	0.903 (0.737- 1.107)	0.328
TC (mmol/L)	0.886 (0.724- 1.084)	0.238
TG (mmol/L)	0.524 (0.375-0.734)	< 0.001
HDL-C (mmol/L)	0.395 (0.225-0.693)	0.001
LDL-C (mmol/L)	0.991 (0.756- 1.298)	0.946
CTRP3 (ng/mL)	1.129 (1.095- 1.165)	< 0.001
CTRP4 (pg/mL)	1.066 (1.050- 1.082)	< 0.001
CTRP14 (ng/mL)	1.183 (1.091- 1.283)	< 0.001

Abbreviations: OR, odds ratio; CI, confidence interval; APOE, apolipoprotein E; BMI, body mass index; TC, total cholesterol; TG, triglyceride; HDL-C, high-density lipoprotein cholesterol; LDL-C,low-density lipoprotein cholesterol; CTRP, C1q/tumor necrosis factor-related protein. $P < 0.05$ is considered the statistical significance.
