# Supplementary material for: Association between plasma CTRPs with cognitive impairment and neurodegeneration of Alzheimer's disease
Source: CNS Neurosci Ther. 2024 Feb 9;30(2):e14606. doi: 10.1111/cns.14606 (PMC10853890; doi:10.1111/cns.14606)
Supplement: Supplementary file 11 — Table S2 [file CNS-30-e14606-s001.pdf]

Supplemental Table 2. Pairwise comparison of the area under the receiver operator characteristic curves by the DeLong's test.

	**Difference between areas**	***Z* statistic**	***p*-value**
CTRP 3 vs. CTRP 4	0.001	0.038	$p = 0.9694$
CTRP 3 vs. CTRP 14	0.176	4.353	$p < 0.0001$
CTRP 3 vs. CTRP 3+4	0.283	10.611	$p < 0.0001$
CTRP 3 vs. CTRP 3+14	0.030	2.485	$p = 0.0130$
CTRP 3 vs. CTRP 4+14	0.272	7.216	$p < 0.0001$
CTRP 3 vs. CTRP 3+4+14	0.273	11.154	$p < 0.0001$
CTRP 4 vs. CTRP 14	0.177	4.549	$p < 0.0001$
CTRP 4 vs. CTRP 3+4	0.284	5.883	$p < 0.0001$
CTRP 4 vs. CTRP 3+14	0.029	1.049	$p = 0.2943$
CTRP 4 vs. CTRP 4+14	0.274	9.256	$p < 0.0001$
CTRP 4 vs. CTRP 3+4+14	0.275	6.137	$p < 0.0001$
CTRP 14 vs. CTRP 3+4	0.107	2.179	$p = 0.0294$
CTRP 14 vs. CTRP 3+14	0.206	6.385	$p < 0.0001$
CTRP 14 vs. CTRP 4+14	0.097	1.554	$p = 0.1202$
CTRP 14 vs. CTRP 3+4+14	0.098	1.740	$p = 0.0818$
CTRP 3+4 vs. CTRP 3+14	0.313	10.825	$p < 0.0001$
CTRP 3+4 vs. CTRP 4+14	0.010	0.193	$p = 0.8473$
CTRP 3+4 vs. CTRP 3+4+14	0.009	0.541	$p = 0.5884$
CTRP 3+14 vs. CTRP 4+14	0.302	7.185	$p < 0.0001$
CTRP 3+14 vs. CTRP 3+4+14	0.303	9.809	$p < 0.0001$
CTRP 4+14 vs. CTRP 3+4+14	0.001	0.024	$p = 0.9813$

Abbreviations: CTRP, C1q/ tumor necrosis factor-related protein. $P < 0.05$ is considered the statistical significance.
